# Evaluation of three commercial assays for SARS-CoV-2 molecular detection in upper respiratory tract samples

**DOI:** 10.1007/s10096-020-04025-0

**Published:** 2020-09-04

**Authors:** Flora Marzia Liotti, Giulia Menchinelli, Simona Marchetti, Grazia Angela Morandotti, Maurizio Sanguinetti, Brunella Posteraro, Paola Cattani

**Affiliations:** 1 Dipartimento di Scienze Biotecnologiche di Base, Cliniche Intensivologiche e Perioperatorie, Università Cattolica del Sacro Cuore, Rome, Italy; 2 Dipartimento di Scienze di Laboratorio e Infettivologiche, Fondazione Policlinico Universitario A. Gemelli IRCCS, Rome, Italy; 3 Dipartimento di Scienze Gastroenterologiche, Endocrino-Metaboliche e Nefro-Urologiche, Fondazione Policlinico Universitario A. Gemelli IRCCS, Rome, Italy

**Keywords:** SARS-CoV-2, COVID-19, Molecular assay, Viral RNA load, Respiratory samples

## Abstract

The increasing COVID-19 widespread has created the necessity to assess the diagnostic accuracy of newly introduced (RT-PCR based) assays for SARS-CoV-2 RNA detection in respiratory tract samples. We compared the results of the Allplex™ 2019-nCoV assay with those of the Simplexa™ COVID-19 Direct assay and the Quanty COVID-19 assay, respectively, all performed on 125 nasal/oropharyngeal swab samples of patients with COVID-19 suspicion. Fifty-four samples were positive, and 71 were negative with the Allplex™ assay, whereas 47 of 54 samples were also positive with the Simplexa™ assay. The Quanty assay detected 55 positive samples, including the 54 positive samples with the Allplex™ assay and 1 sample that was Allplex™ negative but Simplexa™ positive. Using a consensus result criterion as the reference standard allowed to resolve the eight samples with discordant results (one Allplex™ negative and seven Simplexa™ negative) as truly false negative. Interestingly, a Spearman’s negative association was found between the viral RNA loads quantified by the Quanty assay and the *C*_*T*_ values of RT PCRs performed with either the Allplex™ assay or the Simplexa™ assay. However, the strength of this association was higher for the Allplex™ assay (N gene, *ρ* = − 0.92; RdRP gene, *ρ* = − 0.91) than for the Simplexa™ assay (ORF1ab gene, *ρ* = − 0.65; S gene, *ρ* = − 0.80). The Allplex™ 2019-nCoV, the Simplexa™ COVID-19 Direct, and the Quanty COVID-19 assays yielded comparable results. However, the role these assays might play in future clinical practice warrants larger comparison studies.

**Electronic supplementary material:**

The online version of this article (10.1007/s10096-020-04025-0) contains supplementary material, which is available to authorized users.

## Introduction

Since first isolation on December 2019 [1], the severe acute respiratory syndrome coronavirus 2 (SARS-CoV-2)—initially called 2019-nCoV—which causes the illness referred to as coronavirus disease 2019 (COVID-19) has increasingly spread worldwide. By 29 April 2020, the number of confirmed cases reported by the World Health Organization (WHO) had reached 3,023,788 (https://covid19.who.int/), hence representing an unprecedented viral pandemic. To prevent virus transmission and/or ensure appropriate management of COVID-19 patients [2], clinical microbiology laboratories are constantly requested to implement relatively quick and sensitive diagnostic assays for SARS-CoV-2 RNA detection in clinical samples [3].

Nowadays, real-time reverse transcription-polymerase chain reaction (RT-PCR)-based assay performed on upper respiratory tract (URT) samples (e.g., nasopharyngeal and/or oropharyngeal swabs) is the current diagnostic strategy to confirm COVID-19 cases [4], regardless of clinical disease manifestation [5]. In general, diagnosis relies upon the in vitro amplification of one or more molecular targets within the positive-sense, single-stranded SARS-CoV-2 RNA, including the envelope (E), RNA-dependent RNA polymerase (RdRP), and nucleocapsid (N) genes, among others [6, 7]. In particular, the assay developed by the Centers for Disease Control and Prevention (CDC)—the most widely used in the USA—utilizes two N gene regions (N1 and N2) as targets [4].

As soon as the WHO published protocols for RT-PCR assays [8], Seegene launched the Allplex™ 2019-nCoV assay—approved for emergency use authorization (EUA) from US Food and Drug Administration (FDA) on 21 April 2020. This single-tube assay identifies E, RdRP, and N genes, as established by the WHO (https://www.who.int/emergencies/diseases/novel-coronavirus-2019/technical-guidance/laboratory-guidance). Later, DiaSorin Molecular developed the Simplexa™ COVID-19 Direct assay, for which the FDA granted a EUA on 19 March 2020 [4]. The assay targets two regions within the SARS-CoV-2 genome, one encoding the spike (S) protein (i.e., the S gene) and the other well-conserved non-structural proteins (i.e., the open reading frames ORF1a and ORF1b) of SARS-CoV-2. Remarkably, both assays received CE (Conformité Européenne) marking. In parallel, the CE-marked Clonit Quanty COVID-19 assay was developed according to CDC guidelines (https://www.cdc.gov/) to detect and, importantly, quantify SARS-CoV-2 RNA in clinical samples using three N gene regions (N1, N2, and N3) as targets. However, the true sensitivity of currently available assays is unknown [9]. In particular, few studies so far have compared the results obtained with different commercial assays in routine laboratory practice [10–12].

The aim of this study was to perform a comparative evaluation of the Allplex™ 2019-nCoV (Arrow Diagnostics S.r.l., Genova, Italy), the Simplexa™ COVID-19 Direct (DiaSorin Molecular, Saluggia, Vercelli, Italy), and the Quanty COVID-19 (*Clonit* S.r.l, Milan, Italy) assays on nasal/oropharyngeal swab (NOS) samples of patients screened for SARS-CoV-2 infection.

## Materials and methods

### Study design and samples

This retrospective study was performed on NOS samples collected from patients admitted to the Fondazione Policlinico Universitario A. Gemelli (FPG) IRCCS hospital’s emergency department with COVID-19 suspicion during a 2-week period in May 2020. NOS samples were collected together within a single tube of universal transport medium (UTM®; Copan Italia S.p.A., Brescia, Italy) to prevent viral RNA degradation and/or bacterial/fungal overgrowth. We considered all samples tested for SARS-CoV-2 RNA by the Allplex™ 2019-nCoV assay (see below) eligible for inclusion. Among SARS-CoV-2 positive samples, we randomly selected samples that were representative of differing target(s) positive levels, as assessed by their cycle threshold (*C*_*T*_) values (i.e., 17.9–39.4; see also below). We also selected negative samples to reach a number of 125 samples in total. Aliquots of primary samples were immediately frozen and kept at − 70 °C until further analysis. Before testing, aliquots were thawed at room temperature and briefly vortexed.

### SARS-CoV-2 molecular detection

Testing of NOS sample aliquots using SARS-CoV-2 molecular assays was performed in accordance with the manufacturer’s instructions.

#### Allplex™ 2019-nCoV assay

Briefly, 200 μl of sample was processed with a Seegene Nimbus automated system (Arrow Diagnostics), which performs both RNA extraction—using STARMag Universal Cartridge kit—and PCR assay setup. A reaction microplate with therein-extracted RNA was loaded onto a real-time PCR CFX96 Touch™ system (Bio-Rad Laboratories, Hercules, CA, USA). Positive and negative controls were included in each run. After assay’s completion, the Seegene Viewer 2019-nCoV software allowed automated analysis and interpretation of results. A positive result (i.e., a *C*_*T*_ less than 40) for at least one of two viral targets (i.e., RdRP and N genes) or for the E gene alone indicates, respectively, the certain or presumptive presence of SARS-CoV-2 RNA in the patient sample. An invalid result (e.g., due to internal control failure) indicates inconclusive determination of the SARS-CoV-2 RNA presence or absence in the patient sample, thus requiring sample retesting.

#### Simplexa™ COVID-19 Direct assay

Briefly, 50 μl of sample and 50 μl of reaction mixture were separately loaded into Direct real-time PCR amplification-disc wells and onto a LIAISON® MDX instrument (DiaSorin Molecular) and allowed to react for a 75-min run. Positive and negative controls were included in each run. After assay’s completion, the instrument’s Studio software automatically calculated and displayed results. A positive result (i.e., a *C*_*T*_ less than 40) for at least one of two viral targets (i.e., S and ORF1ab genes) indicates the presence of SARS-CoV-2 RNA in the patient sample. As with the Allplex™ 2019-nCoV assay, an invalid result requires sample retesting.

#### Quanty COVID-19 assay

Briefly, separate real-time PCR microplate’s wells were each filled with 5-μl sample’s extracted RNA (i.e., derived from the Nimbus RNA extraction step), positive control, negative control, and standards. For SARS-CoV-2 RNA qualitative detection, the instrument’s software automatically analyzed and interpreted the results. A positive result (i.e., a *C*_*T*_ less than 40) for all three viral targets (N1, N2, and N3 genes) indicates the presence of SARS-CoV-2 RNA in the patient sample. Otherwise, the software defines the result as inconclusive, requiring sample retesting. For SARS-CoV-2 RNA quantitative detection, the software built a standard curve with the *C*_*T*_ values obtained following amplification of the aforementioned standards (which contain 10^1^, 10^2^, 10^3^, 10^4^, and 10^5^ copies/μl of synthetic viral N1-encoding RNA, respectively). This allowed calculating the viral load in the patient sample by interpolation of the corresponding *C*_*T*_ value with the standard curve. Then, the actual viral load of the sample (expressed in copies/ml) was determined multiplying the calculated number of viral copies by 1000/V_e_ and E_v_/E_a_ ratios, where V_e_ is the extracted sample volume (200 μl), E_v_ is the eluted sample volume during the extraction step (100 μl), and E_a_ is the extracted sample volume used for amplification (5 μl). To validate the manufacturer’s standards, we generated a standard curve using the Quantitative Synthetic SARS-CoV2 RNA: ORF, E, and N (ATCC® VR3276SD™), which was diluted at the same concentrations as the standards used in the Quanty COVID-19 assay. In preliminary experiments, each of the ATCC® VR3276SD™ RNA samples was quantified in triplicate with the Quanty COVID-19 assay, and results were in the expected *C*_*T*_ value ranges (data not shown).

### Data analysis

No sample retesting was performed due to the absence of invalid results; consequently, we analyzed the first testing results for all study samples. We calculated sensitivity, specificity, and positive and negative predictive values, together with their respective confidence intervals (CIs), for the Allplex™ 2019-nCoV assay, the Simplexa™ COVID-19 Direct assay, and the Quanty COVID-19 assay. To this end, we used a consensus criterion as the reference standard (i.e., defined as the result obtained from at least two of the three molecular assays) [11]. Analysis was performed with Stata software version 11.1 (StataCorp, College Station, TX, USA). Differences between the *C*_*T*_ values in sample groups were assessed using the Student’s *t* test. Two-sided *P* values of < 0.05 were considered statistically significant. We used Cohen’s kappa to assess the strength of agreement between the assays [13]. Values greater than zero indicated none to slight (0.01–0.20), fair (0.21–0.40), moderate (0.41–0.60), substantial (0.61–0.80), or almost perfect (0.81–1.00) levels of agreement, and values lower than/equal to zero indicated the absence of agreement. To assess the relationship between the viral load levels determined by the Quanty COVID-19 assay and the *C*_*T*_ values determined by the Allplex™ 2019-nCoV or Simplexa™ COVID-19 Direct assays, we performed a Spearman correlation on all samples where the concentration of the SARS-CoV-2 N1 gene was within a range of 10^1^ to 10^7^ copies per ml.

## Results

### Sample positivity by molecular assays

Table 1 depicts the results of 125 NOS samples, which tested either positive (*n* = 54) or negative (*n* = 71) with the Allplex™ 2019-nCoV assay—the first implemented SARS-CoV-2 detection assay in our laboratory. The results were evaluated in comparison with those of the Simplexa™ COVID-19 Direct assay and the Quanty COVID-19 assay. As shown (for details, see Table S1 in the supplemental material), *C*_*T*_ values of Allplex™ 2019-nCoV positive samples ranged from 17.9 to 39.3 for E, RdRP, and N genes (33 samples), 28.4 to 39.3 for RdRP and N genes (9 samples), 33.7 to 39.4 for the N gene (11 samples), and 35.6 to 37.1 for E and N genes (1 sample). In particular, the mean (± SD) *C*_*T*_ value for the E gene (26.4 ± 3.9) was lower than the values for RdRP (28.0 ± 3.6; *P* = 0.09) or N (28.9 ± 4.4; *P* = 0.02) genes in 33 samples and the value for the N gene (37.1) in 1 sample.

**Table 1:** Overall results of 125 NOS samples tested by three molecular SARS-CoV-2 detection assays

	Value for the following assays expressed as number (*C*_*T*_ range)		
	Allplex™ 2019-nCoV	Simplexa™ COVID-19 Direct	Quanty COVID-19
Positive results			
All	54 (17.9–39.4)	48 (17.5–39.7)	55 (18.7–39.8)
By target(s)			
E, RdRP, and N genes	33 (17.9–39.3)		
E and N genes	1 (35.6–37.1)		
RdRP and N genes	9 (28.4–39.3)		
N gene	11 (33.7–39.4)		
S and ORF1ab genes		40 (17.5–39.7)	
S gene		4 (21.0–35.6)	
ORF1ab gene		4 (29.3–34.9)	
N1, N2, and N3 genes			55 (18.7–39.8)
Negative results	71 (0.0–0.0)	77 (0.0–0.0)	70 (0.0–0.0)
No. of concordant results	124	118	117
No. of discordant results	1	7	8

*NOS* nasal/oropharyngeal swab, *C*_*T*_ threshold cycle, *E* envelope, *RdRP* RNA-dependent RNA polymerase, *N* nucleocapsid, *S* spike, *ORF* open reading frame

Forty-seven of 54 positive samples by the Allplex™ 2019-nCoV assay had also positive results with the Simplexa™ COVID-19 Direct assay. The *C*_*T*_ values of positive Simplexa™ COVID-19 samples ranged from 17.5 to 39.7 for S and ORF1ab genes (40 samples), 21.0 to 35.6 for the ORF1ab gene (4 samples), and 29.3 to 34.9 for the S gene (4 samples). In particular, the mean (± SD) *C*_*T*_ value for the S gene (27.9 ± 5.1) equated the value for the ORF1ab gene (27.9 ± 3.9; *P* = 0.99) in 40 samples.

Of eight samples with discordant results, seven samples tested positive with the Allplex™ 2019-nCoV assay (the N gene was detected alone or in combination with E and/or RdRP genes) but negative with the Simplexa™ COVID-19 Direct assay. The remaining one sample tested negative with the Allplex™ 2019-nCoV assay but positive with the Simplexa™ COVID-19 Direct assay (both S and ORF1ab genes were detected). As detailed in Table S1, the mean (± SD) *C*_*T*_ value of the N gene in the seven samples with discordant results was 34.7 ± 5.9, and this value differed from that of the 47 remaining Allplex™ 2019-nCoV positive samples (31.2 ± 5.0; *P* = 0.09).

Fifty-five samples, including Allplex™ 2019-nCoV (*n* = 54) and Simplexa™ COVID-19 (*n* = 48) positive samples, tested positive, and the remaining 70 of 125 samples tested negative for all the N gene regions targeted by Quanty COVID-19 assay. The *C*_*T*_ values of positive Quanty COVID-19 assay samples ranged from 18.7 to 39.8 for N1, N2, and N3 genes.

### Analytic performance of molecular assays

Table 2 depicts the analytical performance of the three molecular assays according to the reference standard, which relied on a consensus assays’ result criterion, as above specified. As shown, sensitivity and negative predictive value (NPV) of the Allplex™ 2019-nCoV assay were 98.2% and 97.2%, respectively, those of the Simplexa™ COVID-19 Direct assay were 87.3% and 90.9%, respectively, and those of the Quanty COVID-19 assay were both 100%. When analyzing the results according to single assay’s targets, we found lower sensitivities and NPVs for RdRP (76.4% and 84.3%, respectively) and E (61.8% and 76.9%, respectively) genes in the Allplex™ 2019-nCoV assay and for both S and ORF1ab (80.0% and 86.4%, respectively) genes in the Simplexa™ COVID-19 Direct assay (Table 2).

**Table 2:** Performances of the Allplex™ 2019-nCoV, Simplexa COVID-19 Direct, and Quanty COVID-19 assays according to a consensus criterion used as the reference standard^a^

Allplex 2019-nCoV results by target				
	E gene	RdRP gene	N gene	Total
No. matched positives	34	42	54	54
No. matched negatives	70	70	70	70
No. Allplex 2019-nCoV misses	21	13	1	1
% sensitivity (95% CI)	61.8 (47.7–74.6)	76.4 (63.0–86.8)	98.2 (90.3–100.0)	98.2 (90.3–100.0)
% specificity (95% CI)	100.0 (94.9–100.0)	100.0 (94.9–100.0)	100.0 (94.9–100.0)	100.0 (94.9–100.0)
% PPV (95% CI)	100.0 (89.7–100.0)	100.0 (91.6–100.0)	100.0 (93.3–100.0)	100.0 (93.3–100.0)
% NPV (95% CI)	76.9 (66.9–85.1)	84.3 (74.7–91.4)	97.2 (90.3–99.7)	97.2 (90.3–99.7)
% agreement	83.2	89.6	99.2	99.2
Cohen’s kappa (95% CI)				0.98 (0.95–1.02)
Simplexa COVID-19 results by target				
	S gene	ORF1ab gene		Total
No. matched positives	44	44		48
No. matched negatives	70	70		70
No. Simplexa COVID-19 misses	11	11		7
% sensitivity (95% CI)	80.0 (67.0–89.6)	80.0 (67.0–89.6)		87.3 (75.5–94.7)
% specificity (95% CI)	100.0 (94.9–100.0)	100.0 (94.9–100.0)		100.0 (94.9–100.0)
% PPV (95% CI)	100.0 (92.0–100.0)	100.0 (92.0–100.0)		100.0 (92.6–100.0)
% NPV (95% CI)	86.4 (77.0–93.0)	86.4 (77.0–93.0)		90.9 (82.2–96.3)
% agreement	91.2	91.2		94.4
Cohen’s kappa (95% CI)				0.88 (0.80–0.97)
Quanty COVID-19 results by target				
	N1 gene	N2 gene	N3 gene	Total
No. matched positives	55	55	55	55
No. matched negatives	70	70	70	70
No. Quanty COVID-19 misses	0	0	0	0
% sensitivity (95% CI)	100.0 (93.5–100.0)	100.0 (93.5–100.0)	100.0 (93.5–100.0)	100.0 (93.5–100.0)
% specificity (95% CI)	100.0 (94.9–100.0)	100.0 (94.9–100.0)	100.0 (94.9–100.0)	100.0 (94.9–100.0)
% PPV (95% CI)	100.0 (93.5–100.0)	100.0 (93.5–100.0)	100.0 (93.5–100.0)	100.0 (93.5–100.0)
% NPV (95% CI)	100.0 (94.9–100.0)	100.0 (94.9–100.0)	100.0 (94.9–100.0)	100.0 (94.9–100.0)
% agreement	100.0	100.0	100.0	100.0
Cohen’s kappa (95% CI)				1.00 (1.0–1.0)

*E* envelope, *RdRP* RNA-dependent RNA polymerase, *N* nucleocapsid, *S* spike, *ORF* open reading frame, *CI* confidence interval, *PPV* positive predictive value, *NPV* negative predictive value

^a^The reference standard was defined as the result obtained from at least two of the three molecular assays under evaluation [11]

### Relationship between samples’ C_T_ values and viral loads

Table 3 shows the viral loads determined by the Quanty COVID-19 assay (expressed as log_10_ N1 copies per ml) in positive samples, which were stratified by the Allplex™ 2019-nCoV (E, RdRP, and N) or the Simplexa™ COVID-19 Direct (S and ORF1ab) assays’ targets. We found highest proportions of E (29.4% and 26.5%, respectively), RdRP (23.8% and 26.2%, respectively), and N (22.2% and 20.4%, respectively) gene detections, as well as S (27.3% and 22.7%, respectively) and ORF1ab (25.0% and 22.7%, respectively) gene detections in samples with viral load levels ranging from > 3.0 to ≤ 4.0 or > 4.0 to ≤ 5.0 log_10_ copies per ml.

**Table 3:** Detection results of Allplex™ 2019-nCoV and the Simplexa™ COVID-19 Direct assays’ targets according to the viral load levels in positive NOS samples, as determined by the Quanty COVID-19 assay

Viral load levels (log_10_ copies/ml)	No. (%) of detections by Allplex 2019-nCoV targets^a^			No. (%) of detections by Simplexa COVID-19 targets^b^	
	E gene	RdRP gene	N gene	S gene	ORF1ab gene
	*n* = 34	*n* = 42	*n* = 54	*n* = 44	*n* = 44
≤ 1.0	0 (0.0)	0 (0.0)	1 (1.9)	0 (0.0)	0 (0.0)
> 1.0–≤ 2.0	3 (8.8)	5 (11.9)	12 (22.2)	7 (16.0)	10 (22.7)
> 2.0–≤ 3.0	2 (5.9)	6 (14.3)	8 (14.8)	6 (13.6)	5 (11.4)
> 3.0–≤ 4.0	10 (29.4)	10 (23.8)	12 (22.2)	12 (27.3)	11 (25.0)
> 4.0–≤ 5.0	9 (26.5)	11 (26.2)	11 (20.4)	10 (22.7)	10 (22.7)
> 5.0–≤ 6.0	7 (20.6)	7 (16.7)	7 (13.0)	6 (13.6)	5 (11.4)
> 6.0–≤ 7.0	3 (8.8)	3 (7.1)	3 (5.5)	3 (6.8)	3 (6.8)

^a^The Allplex 2019-nCoV targets the E (envelope), RdRP (RNA-dependent RNA polymerase), and N (nucleocapsid) genes of SARS-CoV-2

^b^The Simplexa COVID-19 targets the S (spike) and ORF1ab (open reading frame 1ab) genes of SARS-CoV-2

**Fig. 1:**
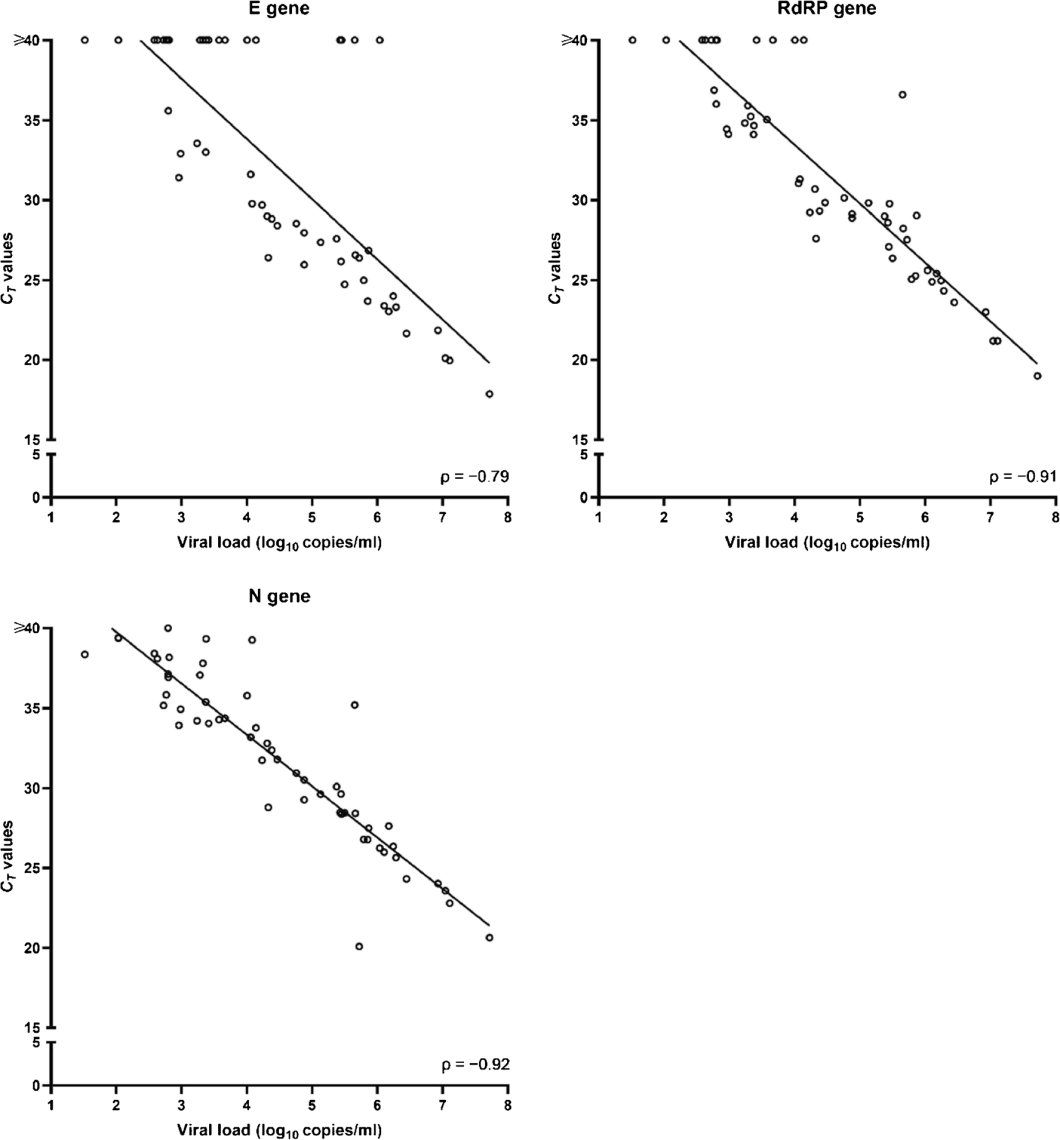
Correlation between the viral load levels quantified by the Quanty COVID-19 assay and the *C*_*T*_ values obtained with the Allplex™ 2019-nCoV assay. Values are shown for each SARS-CoV-2 gene (E, RdRP, or N) detected by the assay

**Fig. 2:**
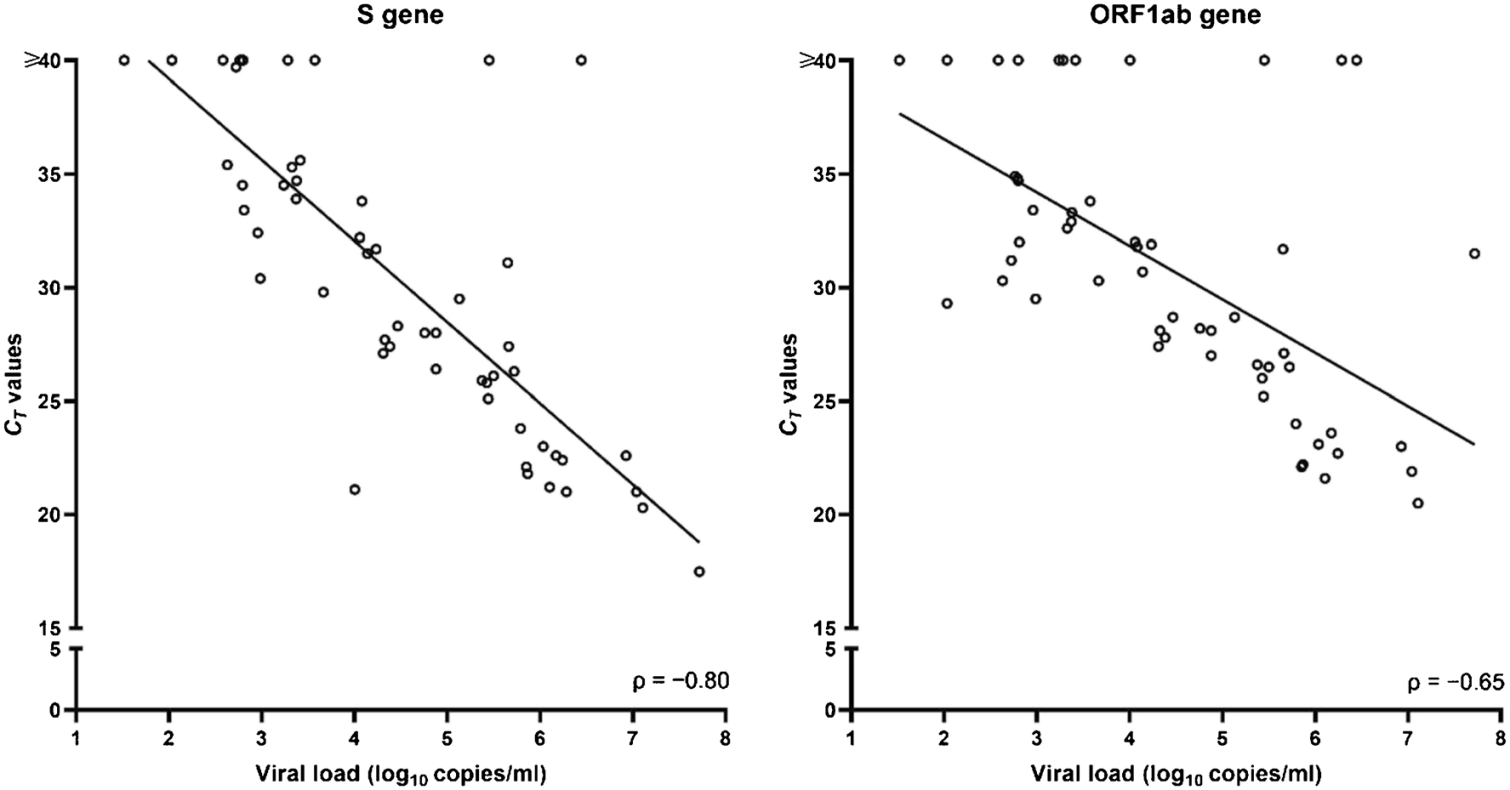
Correlation between the viral load levels quantified by the Quanty COVID-19 assay and the *C*_*T*_ values obtained with the Simplexa™ COVID-19 Direct assay. Values are shown for each SARS-CoV-2 gene (S or ORF1ab) detected by the assay

To determine if there was relationship between viral load and *C*_*T*_ value, we performed a Spearman’s correlation analysis. Before that, samples with *C*_*T*_ values ≥ 40 by the Allplex™ 2019-nCoV assay or the Simplexa™ COVID-19 Direct assay were assigned a value of 40. Analyzing all 55 samples that tested positive or negative by the assays, we found a strong (negative) association between the *C*_*T*_ values of N (Spearman’s *ρ* = − 0.92; *P* < 0.001) and RdRP (*ρ* = − 0.91; *P* < 0.001) genes—detected by the Allplex™ 2019-nCoV assay—and viral loads (Fig. 1). Conversely, we found a less strong (negative) association between the *C*_*T*_ values of ORF1ab (*ρ* = − 0.65; *P* < 0.001) and S (*ρ* = − 0.80; *P* < 0.001) genes—detected by the Simplexa™ COVID-19 Direct assay—and viral loads (Fig. 2).

## Discussion

The current speed with which the laboratory-based diagnostic landscape for COVID-19 is changing [3] creates an impelling necessity to assess rigorously the diagnostic accuracy of newly introduced SARS-CoV-2 assays. The DiaSorin Molecular Simplexa™ COVID-19 Direct assay is one of 28 commercially available assays that was EUA granted from the FDA as of 4 April 2020 [4]. One study compared the DiaSorin Molecular assay with the Abbott ID Now assay, using a modified CDC assay as the reference standard [10]. Another study compared the DiaSorin Molecular assay with a modified CDC Diagnostic Panel, the Diagnostics GenMark ePlex SARS-CoV-2 assay, and the Hologic Panther Fusion SARS–CoV-2 assay [11]. In the latter study [11], the authors used a “consensus result,” namely, a result obtained by at least three out of four evaluated assays, to establish the reference standard. Both the studies tested URT samples (*n* = 96 [10] and *n* = 104 [11], respectively). Using the same criterion [11], we independently assessed the performance of the Simplexa™ COVID-19 Direct assay and the Quanty COVID-19 assay in comparison with that of the Allplex™ 2019-nCoV assay—one of the first commercialized assays since SARS-CoV-2 had been isolated for the first time [1]. Additionally, we used the Quanty COVID-19 assay to quantitate the SARS-CoV-2 RNA (i.e., the N1 gene) in the 125 NOS samples (Table S1) under consideration.

Our findings show that, while the Quanty COVID-19 assay displayed 100% agreement with the reference standard, the Allplex™ 2019-nCoV and the Simplexa™ COVID-19 Direct assays yielded comparable results (99.2% and 94.4%, respectively). Discordant results were found in eight positive samples, i.e., one false negative by the Allplex™ 2019-nCoV assay and seven false negatives by the Simplexa™ COVID-19 Direct assay (sensitivity was 98.2% and 87.3%, respectively). The reasons for the discordant results are unknown. We noticed that the sample testing false negative with the Allplex™ 2019-nCoV assay was true positive with the Simplexa™ COVID-19 Direct assay and had *C*_*T*_ values (34.5 [S gene] and 34.8 [ORF1ab gene]) comparable with those of the Quanty COVID-19 assay (38.3 [N2 gene] and 37.8 [N3 gene]). The viral load in this sample equated to 6.2 × 10^2^ RNA copies/ml, and we found a similar value in other five samples (range, 5.3 × 10^2^ to 6.5 × 10^2^ RNA copies/ml) included in this analysis. Except for one (Simplexa™ COVID-19 negative) sample, these samples tested positive with both the Allplex™ 2019-nCoV (two for N gene alone and two for both N and RdRP genes) and the Simplexa™ COVID-19 Direct (two for ORF1ab gene alone and two for both S and ORF1ab genes) assays. The viral loads of seven samples with a false-negative result by the Simplexa™ COVID-19 Direct assay ranged from 3.3 × 10^1^ to 2.8 × 10^6^ RNA copies/ml, and three of these samples were under the limit of detection estimated as 500 RNA copies/ml (https://www.molecular.diasorin.com) or reported as 16 to 62 RNA copies/ml [11] for the DiaSorin Molecular assay. Thus, the false negativity observed, particularly with the Simplexa™ COVID-19 Direct assay, might not be due to a scarce copy number of SARS-CoV-2 RNA in those samples. Consequently, we could not rule out that intrinsic reasons (e.g., virus mutation) have affected the RT-PCR result in our samples. Unfortunately, we did not perform viral sequencing to clarify this issue [14].

To reduce the potential risks of cross-reactions with endemic (HCoV-229E, HCoV-NL63, HCoV-OC43, and HCoV-HKU1) or other epidemic (SARS-CoV and MERS-CoV) coronaviruses and SARS-CoV-2 genome mutations, experts advise to include at least two molecular targets when developing a SARS-CoV-2 detection assay [4]. From the Allplex™ 2019-nCoV assay’s implementation [15] to current use in our laboratory, Seegene modified the interpretative criteria, so that positivity for one of three assay targets is now sufficient to adjudicate a sample as positive for SARS-CoV-2 RNA. Excluding one sample (negative for all three targets), it is remarkable that in all 54 Allplex™ 2019-nCoV positive samples, the N gene was detected. Thus, we are not surprised that the US CDC recommended the N gene as a SARS-CoV-2 assay target alone [16], as well as the N gene was the sole molecular target in the Quanty COVID-19 assay.

As viral dynamics in COVID-19 cases is not fully understood [17], SARS-CoV-2 loads determined by RT PCR assays may not be useful to indicate disease severity [18–20]. However, the viral load in a clinical (primarily URT) sample may be an indication of pathogen transmissibility [21] and correlates with the virus isolation in cell culture [22]. Consistent with studies showing that lower *C*_*T*_ values are inversely related to higher viral copy numbers [19, 20, 23], we found that viral loads were negatively associated with the *C*_*T*_ values of RT PCRs performed with either the Allplex™ 2019-nCoV assay or the Simplexa™ COVID-19 Direct assay. However, we noted a slight difference in the strength of this association between assays, which was in favor of the Allplex™ 2019-nCoV assay. As the Simplexa™ COVID-19 Direct assay was performed on frozen samples whereas the Allplex™ 2019-nCoV assay on fresh samples, we do not exclude the possibility of viral RNA degradation by freezing, which might have lowered the viral loads in the samples tested with the Simplexa™ COVID-19 Direct assay. Otherwise, the fact that the SARS-CoV-2 N gene is not targeted by the Simplexa™ COVID-19 Direct assay could explicate the low association between *C*_*T*_ values and viral loads seen with this assay.

While confirming previously published results (albeit restricted to the Simplexa™ COVID-19 Direct assay) [10, 11], we expanded the general knowledge about performance features of commercially available molecular SARS-CoV-2 detection assays (including sample-to-answer platforms [24–26]). The finding that one molecular target would work better than the other is helpful in redesigning such assays (e.g., shifting from multiple targets to a single target) to enhance reagent utilization [3]. Meanwhile, showing the equivalence of assays may aid to promptly redirect our laboratory choice of RNA-based diagnostic assays towards those with less supply chain trouble at that time [3]. Compared with the Allplex™ 2019-nCoV assay or the Quanty COVID-19 assay, the Simplexa™ COVID-19 Direct assay has the advantage of quicker turnaround test results (75 min vs 4–5 h, respectively). Because the time to perform test is an important criterion, use of the Simplexa™ COVID-19 Direct assay instead of Allplex™ 2019-nCoV assay or the Quanty COVID-19 assay should be favored. However, in the case of a massive crisis such as the one we experienced, working on 96-well plates for RNA extraction and RT-PCR for a 4–5 h duration can be time saving, compared with a test that allows to obtain results in 75 min but at low output (i.e., with a 1–8 sample format).

In conclusion, the study showed that the Allplex™ 2019-nCoV assay is equivalent to the Simplexa™ COVID-19 Direct assay for the laboratory-confirmed diagnosis of COVID-19, whereas the Quanty COVID-19 assay allows to maximize diagnosis. Additionally, the Quanty COVID-19 assay providing quantitative data may be useful for SARS-CoV-2 infection monitoring purposes. However, further studies are warranted to define the role these assays might play in future clinical practice. Certainly, as testing for COVID-19 increases, these assays or their refinements will contribute to improve the laboratory capacity to identify patients with SARS-CoV-2 infection.

## Electronic supplementary material

ESM 1 (DOCX 32 kb)
