# Supplementary material for: Evaluation of three commercial assays for SARS-CoV-2 molecular detection in upper respiratory tract samples
Source: Eur J Clin Microbiol Infect Dis. 2020 Sep 4;40(2):269–77. doi: 10.1007/s10096-020-04025-0 (PMC7471581; doi:10.1007/s10096-020-04025-0)
Supplement: Supplementary file 1 — (DOCX 32 kb) [file 10096_2020_4025_MOESM1_ESM.docx]

**Table S1**

Description of NOS samples that tested positive by the molecular assays used in the study.^a^

| Sample designation | Results (expressed as *C_T_* or copies/ml) by each assay’s targets: | | | | | | | |
| --- | --- | --- | --- | --- | --- | --- | --- | --- |
|  | Allplex™ 2019-nCoV assay | | | Simplexa™ COVID-19 Direct assay | | Quanty COVID-19 assay | | |
|  | E gene | RdRP gene | N gene | S gene | ORF1ab gene | N2 gene | N3 gene | N1 gene |
| 1 | – | – | 38.37 | – | – | 39.86 | 37.84 | 3.3 × 10^1^ |
| 2 | 26.40 | 27.60 | 28.80 | 27.70 | 28.10 | 29.48 | 30.21 | 2.1 × 10^4^ |
| 3 | – | 36.60 | 35.20 | 31.10 | 31.70 | 34.11 | 34.82 | 4.5 × 10^2^ |
| 4 | 24.28 | 25.60 | 26.24 | 23.00 | 23.10 | 26.30 | 26.48 | 1.1 × 10^6^ |
| 5 | – | 35.24 | 37.81 | 35.30 | 32.60 | 34.37 | 36.13 | 2.1 × 10^3^ |
| 6 | – | – | 38.19 | 33.40 | 32.00 | 34.14 | 34.24 | 6.5 × 10^2^ |
| 7 | – | – | 38.43 | – | – | 35.38 | 35.87 | 3.8 × 10^2^ |
| 8 | – | – | 34.05 | 35.60 | – | 32.85 | 32.13 | 2.6 × 10^3^ |
| 9 | – | 35.04 | 34.28 | – | 33.80 | 32.41 | 31.87 | 3.8 × 10^3^ |
| 10 | 25.96 | 28.88 | 29.27 | 26.40 | 27.00 | 28.37 | 28.10 | 7.5 × 10^4^ |
| 11 | – | 36.02 | 36.94 | – | 34.70 | 37.38 | 35.15 | 6.3 × 10^2^ |
| 12 | 31.40 | 34.45 | 33.93 | 32.40 | 33.40 | 34.57 | 34.32 | 9.1 × 10^2^ |
| 13 | 33.55 | 34.83 | 34.21 | 34.50 | – | 34.66 | 34.14 | 1.7 × 10^3^ |
| 14 | 23.03 | 25.42 | 27.63 | 22.60 | 23.60 | 25.46 | 25.22 | 1.5 × 10^6^ |
| 15 | 17.87 | 19.00 | 20.65 | 17.50 | 31.50 | 18.72 | 19.28 | 5.2 × 10^7^ |
| 16 | 28.40 | 29.84 | 31.79 | 28.30 | 28.70 | 30.21 | 30.24 | 2.9 × 10^4^ |
| 17 | 27.96 | 29.12 | 30.50 | 28.00 | 28.10 | 29.09 | 29.34 | 7.5 × 10^4^ |
| 18 | 23.39 | 24.89 | 25.99 | 21.20 | 21.60 | 24.69 | 25.08 | 1.3 × 10^6^ |
| 19 | 23.30 | 24.32 | 25.65 | 21.00 | – | 24.20 | 24.81 | 1.9 × 10^6^ |
| 20 | – | – | 35.78 | 21.10 | – | 31.45 | 33.77 | 1.0 × 10^4^ |
| 21 | – | – | 38.11 | 35.40 | 30.30 | 36.48 | 37.69 | 4.3 × 10^2^ |
| 22 | – | – | 39.40 | – | 29.30 | 37.18 | 37.35 | 1.1 × 10^2^ |
| 23 | – | – | 35.17 | 39.70 | 31.20 | 35.05 | 34.41 | 5.3 × 10^2^ |
| 24 | – | 34.66 | 39.33 | 34.70 | 33.30 | 32.93 | 32.57 | 2.4 × 10^3^ |
| 25 | – | – | 39.37 | – | – | 38.36 | 38.69 | 1.1 × 10^2^ |
| 26 | 28.83 | 29.31 | 32.37 | 27.40 | 27.80 | 29.45 | 28.74 | 2.4 × 10^4^ |
| 27 | 33.00 | 34.11 | 35.39 | 33.90 | 32.90 | 33.61 | 33.97 | 2.3 × 10^3^ |
| 28 | 19.96 | 21.18 | 22.79 | 20.30 | 20.50 | 22.13 | 22.29 | 1.3 × 10^7^ |
| 29 | 20.10 | 21.18 | 23.57 | 21.00 | 21.90 | 22.14 | 21.98 | 1.1 × 10^7^ |
| 30 | 21.85 | 22.99 | 24.02 | 22.60 | 23.00 | 23.88 | 24.01 | 8.4 × 10^6^ |
| 31 | 28.99 | 30.69 | 32.80 | 27.10 | 27.40 | 32.64 | 32.08 | 2.0 × 10^4^ |
| 32 | 21.65 | 23.60 | 24.31 | – | – | 24.21 | 24.07 | 2.8 × 10^6^ |
| 33 | – | – | – | 34.50 | 34.80 | 38.35 | 37.86 | 6.2 × 10^2^ |
| 34 | – | – | 34.37 | 29.80 | 30.30 | 34.38 | 34.01 | 4.6 × 10^3^ |
| 35 | 23.68 | 25.26 | 26.78 | 22.10 | 22.10 | 26.04 | 26.25 | 7.1 × 10^5^ |
| 36 | 32.91 | 34.13 | 34.94 | 30.40 | 29.50 | 35.89 | 36.16 | 9.6 × 10^2^ |
| 37 | 35.59 | – | 37.13 | – | – | 38.08 | 37.21 | 6.3 × 10^2^ |
| 38 | 24.73 | 26.36 | 28.46 | 26.10 | 26.50 | 27.33 | 27.33 | 3.1 × 10^5^ |
| 39 | 28.53 | 30.13 | 30.94 | 28.00 | 28.20 | 31.37 | 31.33 | 5.7 × 10^4^ |
| 40 | – | 36.89 | 35.83 | – | 34.90 | 36.79 | 36.34 | 5.8 × 10^2^ |
| 41 | 26.57 | 28.23 | 28.42 | 27.40 | 27.10 | 28.76 | 28.99 | 4.6 × 10^5^ |
| 42 | 26.16 | 27.08 | 29.64 | 25.10 | 25.20 | 29.19 | 29.08 | 2.8 × 10^5^ |
| 43 | 23.99 | 24.96 | 26.35 | 22.40 | 22.70 | 26.75 | 26.85 | 1.7 × 10^6^ |
| 44 | 27.59 | 28.99 | 30.09 | 25.90 | 26.60 | 29.57 | 29.74 | 2.4 × 10^5^ |
| 45 | 26.38 | 27.53 | 20.09 | 26.30 | 26.50 | 28.52 | 28.15 | 5.3 × 10^5^ |
| 46 | 29.77 | 31.30 | 39.26 | 33.80 | 31.80 | 31.56 | 31.79 | 1.2 × 10^4^ |
| 47 | – | – | 33.77 | 31.50 | 30.70 | 33.57 | 33.22 | 1.4 × 10^4^ |
| 48 | – | 28.60 | 28.47 | 25.80 | 26.00 | 29.05 | 28.91 | 2.7 × 10^5^ |
| 49 | – | 29.77 | 28.38 | – | – | 29.40 | 29.61 | 2.8 × 10^5^ |
| 50 | 24.98 | 25.05 | 26.79 | 23.80 | 24.00 | 27.63 | 27.16 | 6.2 × 10^5^ |
| 51 | 29.69 | 29.22 | 31.74 | 31.70 | 31.90 | 33.27 | 32.73 | 1.7 × 10^4^ |
| 52 | 27.36 | 29.82 | 29.62 | 29.50 | 28.70 | 30.12 | 30.20 | 1.4 × 10^5^ |
| 53 | – | 35.92 | 37.08 | – | – | 36.34 | 35.45 | 1.9 × 10^3^ |
| 54 | 31.60 | 31.04 | 33.18 | 32.20 | 32.00 | 33.38 | 33.02 | 1.1 × 10^4^ |
| 55 | 26.85 | 29.03 | 27.49 | 21.80 | 22.20 | 28.76 | 28.87 | 7.4 × 10^5^ |

^a^NOS, nasal/oropharyngeal swab; *C_T_*, threshold cycle; E, envelope; RdRP, RNA-dependent RNA polymerase; N, nucleocapsid; S, spike; ORF, open reading frame.
